# Experimental Design Supported Liposomal Aztreonam Delivery: *In Vitro* Studies

**DOI:** 10.34172/apb.2021.074

**Published:** 2020-09-15

**Authors:** Sayani Bhattacharyya, Preethi Sudheer, Kuntal Das, Subhabrata Ray

**Affiliations:** ^1^Krupanidhi College of Pharmacy, Bengaluru, Karnataka 560035, India.; ^2^Dr. BC Roy College of Pharmacy, Durgapur, West Bengal 713206, India.

**Keywords:** Aztreonam, Liposomes, Statistical optimization, Antimicrobial study, Cell uptake study

## Abstract

***Purpose:*** The present study focuses on a systemic approach to develop liposomal aztreonam as a promising dosage form for inhalation therapy in the treatment of pneumonia and explores the *in-vitro* antimicrobial and cell uptake efficacy.

***Methods:*** Liposomes were prepared by ethanol injection method using the lipids - soya phosphatidylcholine (SP) and cholesterol (CH). A central composite design (CCD) was employed to optimize the lipid composition to evaluate the effect on vesicle size, zeta potential and entrapment efficiency of the formulation. A numerical and graphical optimization was carried out to predict the optimized blend. The optimized formulation was characterized for vesicle size, surface charge, encapsulation, surface morphology, differential scanning calorimetry (DSC), powder X Ray Diffraction (PXRD), thermogravimetric analysis (TGA), *in vitro* diffusion, accelerated stability studies, antimicrobial studies on *Pseudomonas aeruginosa* NCIM 2200 and *in vitro* cell uptake studies.

***Results:*** The optimized formulation was found to have a particle size of 144 nm, a surface charge of -35 mV, with satisfactory drug entrapment. The surface morphology study proved the formation of nanosized vesicles. The drug release from liposomal matrix was biphasic in nature. The solid-state study revealed the reason for good encapsulation of drug. The moisture retention capacity was found to be minimum. The anti-microbial study revealed the potential antibacterial activity of the optimized formulation over the pure drug. The formulation was found to be safe on the epithelial cells and showed a marked increase in cellular uptake of aztreonam in a lipid carrier.

***Conclusion:*** It can be concluded that the optimized liposomal aztreonam could be considered as a promising approach for the delivery of aztreonam through inhalation.

## Introduction

Lung disease in infants and elderly people has been recognized to be the greatest reason for morbidity and mortality.^1^ Chronic infection in lungs leads to acute exacerbations and affects the functioning of the lungs. *Pseudomonas aeruginosa*, a gram-negative bacterium is considered as the most important pathogen in infections.^2^ It is an opportunistic bacterium that colonizes in the bronchopulmonary tract and forms a thick biofilm owing to its fast growth.^3^ Antimicrobial resistance has also been reported to the class of antibiotics known as carbapenems.^4^ The low permeability of the bacterial outer membrane, efflux mechanisms and the synthesis and secretion of various enzymes by the bacteria made antibacterial agents difficult to permeate through the cell membrane of the bacteria. Hence the development of bacterial resistance is quite frequent.^5^

A review of literature showed that antibiotic loaded liposomes had better activity against *P. aeruginosa* compared to its free drug.^6^ Microorganisms responsible for pneumonia exhibit a high intrinsic resistance because of its outer membrane which restricts low permeation to antimicrobials. Liposomal formulations are found to be effective to fuse with the outer membrane of bacteria and thus can penetrate easily through the cell wall or biofilm produced by them. Further such liposomal formulations are found to be effective in mucus penetration, bypassing pulmonary clearance. Liposomes also help to maintain a steady state drug concentration to inhibit biofilm growth. This quality is attributed to its release profile which happens in two phases, with a preliminary launch followed by sustained release of the drug.^7^

Rudokas et al^8^ reviewed on pulmonary delivery of liposomal anti-cancer drugs through nebulization therapy and their effect of composition and liposome size on therapeutic outcomes. They elaborated on the success of liposomes as an inhalable carrier for the treatment of other lung diseases. Shirley^9^ studied on liposomal inhalation suspension of amikacin (ALIS; Arikayce®) to facilitate targeted and localized drug delivery of drugs, minimizing systemic exposure. They reported that the ALIS was a useful option for the treatment of *Mycobacterium* disease over to conventional dosage form.

Aztreonam belongs to the class of synthetic monobactam, which shows activity against gram-negative aerobic organisms. It inhibits bacterial cell wall synthesis and is stable to most β-lactamases. It is reported to produce a significant effect when combined with aminoglycosides in the treatment of infection with *P. aeruginosa.*^4^ Aztreonam is poorly absorbed orally and the biological half-life is 1.7 hours, which makes it a poor candidate for a conventional delivery system.^10^ Cayston, a reconstituted inhalation solution of lyophilized aztreonam is available in the market for fast nebulization for patients with cystic fibrosis with flexible dosing i.e., 3 times daily upto 28 days.^11^ This new form of delivery offered less systemic exposure of the drug with minimal side effects compared to parenteral administration.

Therefore, the present study focuses on the development of aztreonam loaded liposomal formulation suitable for inhalation with a biphasic release with more retention time in lungs, overcoming adaptive resistance, and minimizing the distribution of the drug to other organs and tissues with associated reductions in its potential toxicities.^12^ The composition of lipids plays an important role in the formulation aspects and stability of liposomes.^13^ The objective of the present study is to predict an optimum composition and liposome size through a statistical optimization process and evaluate different physiochemical properties of the optimized formulation. A liposomal inhalation suspension of aztreonam will be a better option to provide site specific release with a prolonged pharmacological response, by ensuring higher penetrability and long residence time against biofilm growth during infection. The authors focused on formulation development and *in-vitro* evaluation of liposomal aztreonam as a promising mode of delivery for inhalation therapy.

## Materials and Methods

The lipids, soya phosphatidylcholine (SPC) and cholesterol (CH) were obtained from Sigma Aldrich. Aztreonam was gifted by FUAN pharmaceutical group Chongqing Bosen Pharmaceutical Co. Ltd., China. All the other chemicals were of analytical grade. Ethanol was purchased from Qualigens, India. Double distilled water was used for the experimentation.

### Chromatographic determination of aztreonam

For the quantification of aztreonam high performance liquid chromatography (HPLC) method was established in Phenomenex (USA) instrument, with UV SPD10A detector at 300 nm. Chromatographic separation was carried out in a C-8 Phenomenex HPLC column (Dimension 250X4.6 mm, 5 µg). The injection volume and a flow rate were set at 20 µL and 1 mL/min, respectively. The mobile phase was constituted with methanol: phosphate buffer (70:30). The apparent pH of the mixed solvent system was adjusted to 5.4 ± 0.1.^14^

### Preparation of liposomes by ethanol injection method Experimental design

A central composite design (CCD) was used to establish the effect of lipid composition on the vesicle size and its distribution, zeta potential and entrapment efficiency of liposomes, using Design-Expert software V11. The proportions of lipids influence the liposomes property and stability. Review of articles revealed that the stable liposomes can be formulated at a SPC:CH molar ratio within 1:1 to 2.^13,15^ Hence in the proposed design the amount of SPC and CH was set in such a way so that the lipid composition was varied in a wide range as mentioned in Table 1. The effect of lipid composition on vesicular size, surface charge and drug entrapment was studied as responses. The design content 11 experimental runs with three center points varied at two levels. The axial points are in -α (-1.414) to + α (+1.414) at a distance outside the low and high level, whereas the center points help to determine the experimental or pure error due to nonlinearity in the responses. After the experimental runs the responses were evaluated to find the model fit and model prediction power.

**Table 1:** Factors and their levels for the CCD model

**Components**	**-1.414**	**-1**	**0**	**+1**	**+1.414**
CH	1	1.15	1.5	1.85	2
SPC	1	1.44	2.5	3.56	4

### Method of liposome preparation

The aztreonam loaded liposomes were prepared by dissolving the required amount of lipids (calculated in molar weights as per CCD design) in a sonicator bath at 40±2°C. A specified amount of drug (75 mg) was dissolved in phosphate citrate buffer pH- 3. The organic phase to aqueous phase ratio was maintained at 0.4. The organic phase was injected using a syringe pump into the drug solution at a rate of 0.6 mL/min. The entire process was carried out under magnetic stirring.^16^ Formation of liposomes occurred when ethanolic solution encountered the aqueous phase and opalescence of the dispersion confirmed the formation of liposomes. This process was assisted by stirring at room temperature for 15 minutes. The dispersion was subjected to rotary evaporation under reduced pressure to remove ethanol.^17^ The non-encapsulated drugs were removed by ultrafiltration Amicon® Ultra-15 Centrifugal Filter Units (Mumbai, India) at 30000 g for 30 minutes. Finally, the buffer was added to resuspend the liposomes and the final dispersion was taken for evaluation.

### Evaluation of liposomes

#### Particle size analysis

Horiba SZ-100 (Retsch Technology GmbH, Germany) nanoparticle dynamic light scattering (DLS) system was used to measure the particle size of liposomes. The samples were ultrasonicated for 20 minutes. The analysis was carried out at about 25°C after proper dilution with double distilled water. Three trials were made for each sample.^18^

### Determination of surface charge

The surface charge (zeta potential) of the experimental formulations was analyzed Horiba SZ-100 (Retsch Technology GmbH, Germany). The formulation was diluted with double distilled water and then measured by Zetasizer. Three trials were made for each sample.^18^

### Determination of entrapment efficiency

This property of liposomes was determined using ultracentrifugation technique in REMI RM12C laboratory centrifuge, Mumbai, India. A definite volume (0.5 mL) of liposomal suspension was centrifuged in at 12000 × g at 25º C for 10 minutes. The supernatant layer was collected, filtered through 0.22 µm AST works disposable syringe filter and suitably diluted to determine the free drug concentration (F_Drug_). In another tube, an equal volume of the liposomal suspension was dissolved and disrupted in an equal volume of ethanol using an ultrasound sonicator (HIC-CD-4820, INDOSATI, Hyderabad, India) bath for 5 minutes, diluted suitably to determine the total amount of drug in the liposome (T_Drug_). All the samples were filtered through 0.22 µm AST works disposable syringe filter. The samples were analyzed through the developed HPLC method and the entrapment efficiency was calculated.^19,20^

Entrapment efficiency was determined using the formula

$$\%Entrapment=\frac{(T_{Drug}-F_{Drug})100}{T_{Drug}}$$

### Optimization of the experimental design

Evaluation of the 11 trial runs was indicative of the effect of variable quantities of SPC and CH on the particle size, entrapment efficiency and zeta potential of the formulations. Therefore, the optimization of the quantity of SPC and CH for the liposome was carried out using Design-Expert® software version 11. The model validation for each response was done at *P*  <  0.05 through fit statistics with regression analysis. A connection between independent and dependent variables was generated by response surface regression analysis using the software.^21^ An optimized formulation (Fopt) was prepared and evaluated for further studies.

### Morphology study

The surface imaging of the optimized formulation was done by transmission electron microscopy (TEM) images using a FEI-Titan-themis (USA). A carbon-coated copper grid was used to hold the diluted liposomal suspension. The sample Fopt was air dried for 12 hours followed by desiccation in a vacuum desiccator. This left a thin liquid film over the holes of copper grid and was taken for observations.^22^

### Atomic force microscopy (AFM) study

AFM was conducted using an atomic force microscope (Park systems, NX-10 AFM). Sample of AFM was prepared by dilution in deionized water followed by sonication. The sample was spread on glass coverslips, and air dried. Topography images were recorded after analyzing the dimensions of the particles in nanoscale in the 3-D and 2-D axis.

### Freeze drying of the optimized sample

The optimized formulation (Fopt) was freeze-dried using Remi freeze-dryer (BK -FD10, India). Using a conservative freeze-drying protocol, the liposomal suspension was mixed with 15% w/v mannitol as lyoprotectants and 5% w/v dextrose as co-lyoprotectants.^23,24^ Samples were placed on the glass petridish on the shelf. Pre freezing was carried out at -40ºC for 24 hours. A reduction of shelf temperature at a rate of 1ºC/min was done to maintain a temperature of -70ºC for 12 hours to obtain complete freezing of the liposomes.

### Differential scanning calorimetry (DSC) study

DSC analysis of the samples was carried out at nitrogen atmosphere (Flow rate of 100 mL/min) with a controlled heating at a rate of 10°C per minute from 30 to 350°C. An empty aluminum pan was used as a reference platform. The thermograms were recorded with the flow of heat.^25^ The pure drug, a blank formulation and a drug loaded optimized lyophilized formulations were studied in the above method.

### Powder X-ray diffraction (PXRD) study

PXRD diffraction pattern of the pure drug and Fopt were studied in Bruker D8 diffractometer instrument. A Cu K-α 1 tube was used as the source. The instrument was set at 40 kV and 30 mA at room temperature. A Scan was carried out from 5 to 60°C of 2 θ at scan rate of 2.77° per minute. The diffraction patterns were recorded for further analysis of the reflection angle and peak intensity.^26^

### Thermogravimetric analysis

TGA of the optimized sample (Fopt) was carried out using DTG 60, Schimadzu, Japan. A measured quantity of sample was taken in platinum pan. Nitrogen flow rate was maintained at 100 mL/min during the experimentation. Heating was carried out at a rate of 10°C per minute from 30 to 700°C. The percentage weight loss was monitored for the freshly prepared optimized sample and the same sample after 3 months.^26^

### *In-vitro* diffusion studies

The optimized sample was tested for *in vitro* diffusion in simulated lungs fluid (Gamble solution pH 7.4) using 0.22 µm membrane filter in Franz diffusion cell.^27^ The diffusion cell consisted of a receptor compartment of 40 mL and a donor compartment of surface area 1.41 cm^2^. The membrane was soaked overnight in the gamble solution prior use. The diffusion process was carried out for the formulation Fopt equivalent to 50 mg of drug. The temperature of the experimental setup was maintained at 37 ± 0.5°C with magnetic stirring at 100 rpm. At a regular interval 1 ml sample was withdrawn, refilled with same volume of gamble solution. The study was continued for 8 hours.^28^ The samples were estimated for drug release at a wavelength of 293 nm in a UV-Visible spectrophotometer.^29^ Three trials were made for determining the release of aztreonam from liposomes.

### Antimicrobial assay

The gram-negative bacterium such as *P. aeruginosa* NCIM 2200was procured from National Centre Institute of Microbiology, Mysore India. The bacterium was restored on nutrient agar medium at 37ºC for 24 hours and stocked at 4ºC on nutrient agar slant. Each time subculture for the bacterium was prepared form the stock before the assay.

### Determination of minimum inhibitory concentration (MIC)

Antimicrobial activities of the Fopt against *P. aeruginosa* NCIM 2200 were studied. Broth dilution technique was used to determine MIC. Bacterium from fresh culture was inoculated in soybean casein digest medium. The formulation Fopt in varied dilutions (400 to 1000 μg/mL) was added to the nutrient broth, containing standardized test organisms of bacterial cells. A turbidity standard (3.0 × 10^9^cfu/mL) was inoculated in each test tube by serial dilution method. A diluted volume of 1ml of liposomal formulation was mixed with 9 mL of nutrient. The tubes were subjected to incubation at 37^o^C for 24 hours. Positive control tubes and negative control tubes were prepared to minimize the error. Each test was performed in triplicates to ensure reproducibility.^30,31^

### Determination of zone of inhibition

Agar well diffusion method was used to assess the antibacterial activity of the optimized liposomal formulation. The isolated microbes were subcultured on the specific media at 35-37°C for 24 hours. Discs measuring 6 mm were impregnated with sterile cock borer. A concentration of 50 and 100 μg/mL of optimized preparation was placed in the wells of agar plates. A concentration of 50 μg/mL of aztreonam in distilled water was used as standard. The plates were incubated at 37ºC for 24 hours. The antibacterial activity was recorded by measuring the zone of inhibition (mm) by using sliding calipers.^32,33^ All measurements were done in triplicate.

### In-vitro cell viability and uptake study

For the *in-vitro* cell line study the epithelial cells from CHO *Cricetulus griseus* were cultivated in - Dulbecco’s Modified Eagle Medium with High Glucose (DMEM HG) + F12 medium (1:1) supplemented with 10% fetal bovine serum in an incubator at 37^o^C and 5% CO_2_ atmosphere for 24 hours.

### Cytotoxicity test

Cytotoxicity of each test compound was examined by MTT assay. Cells were trypsinized, aspirated and centrifuged at 300 × g. The DMEM HG medium was used to adjust the cell count in such a way so that 10 000 cells were present in 200 μL of suspension. The cell suspensions with different dilution of liposomal solution and drug were incubated in microtiter plate at 37°C and 5% CO_2_ atmosphere for 24 hours. After incubation, plates were treated with medium containing 10% MTT reagent, and was further incubated at 37°C and 5% CO_2_ atmosphere for 3 hours. The culture medium after addition of DMSO, converted the insoluble purple formazan into a colored solution, which was quantified at 570 nm by a spectrophotometer. From the absorbance data, percentage growth inhibition and half maximum inhibitory concentration for cell growth (IC50) was calculated from the dose-response curve of the cell line.^34,35^

### Cell uptake study

The epithelial cells were treated separately with the safest concentration of reconstituted liposomal solution determined after the evaluation of cytotoxicity study of the liposomal aztreonam. Incubation of the cells was done at 37°C and 5% CO_2_ atmosphere for 24 hours. The cells were aspirated and washed with DPBS. 1.25 mL Lysis buffer containing 1% Triton X - 100 in DPBS was used to lyse the cells. To remove the cell debris the cell lysate was centrifuged at 3000 rpm for 5 minutes.^36,37^ The cell lysate was estimated for the presence of aztreonam by the developed HPLC method to estimate the uptake of the drug by the cells, an untreated cell lysate was used as a blank for the estimation.

### Stability studies

Accelerated stability studies were performed according to the ICH guidelines Q1A (R2) 2003.^38^ The lyophilized sample was stored in a glass vial with rubber closure at room temperature at 25°C ± 2°C/65% RH ± 5% RH for three months. At an interval of one month the samples were diluted with HPLC grade water and were evaluated for particle size, zeta potential and entrapment efficiency.^27^

## Results and Discussion

### Physicochemical evaluation of liposomes and statistical optimization

The purpose of the study was to develop a formulation capable of delivering aztreonam in the alveolar region of the lungs through inhalation with high degree of penetration, and long residence time with a biphasic release profile. Therefore, the study was designed to develop a liposomal product of aztreonam of desired properties. For lungs targeting the most important factors to consider is the particle size. Particle size of less than 0.5 µm can reach the acinar region of the lungs.^39^ The electrical charge on the surface influence the surface deposition and effectiveness of the device.^40^ The entrapment of the drug in the lipid bilayer was also a prime concern.

The CCD uniform precision method constituted an effective approach for optimization of liposomal formulations with a wide variety of experimental choices as described in the methodology. The experimental results of the eleven formulations showed a large variation on responses like particle size, % drug entrapment efficiency and zeta potential in the range of 97±3.05 to 361±1.04 nm, 53±0.05 to 75±0.03% and -39±1.3 to -25±1.3 mV respectively as shown in Table 2. The polydispersity index of the particles varied from 0.129 to 0.73.

**Table 2:** Experimental design with observed responses

**Formulation Code**	**SPC**	**CH**	**Mean Particle size (nm)**	**%Entrapment Efficiency**	**Zeta Potential (mV)**
F1	-1	1	133±8.1	68±0.05	-28.2±3.2
F2	0	0	182±8.8	68±0.05	-36±4.1
F3	1	-1	102±3.8	64±0.02	-31.8±2.2
F4	0	0	180±4.34	75±0.03	-37±1.5
F5	-1	-1	117±3	68±0.03	-28±1.2
F6	0	-1.41421	173±2.05	55±0.03	-28±2.1
F7	0	1.41421	170±1.12	53±0.05	-25±1.3
F8	0	0	184±2.14	70±0.05	-39±1.3
F9	-1.41421	0	97±3.05	71±0.03	-27.3±2.4
F10	1.41421	0	223±2.01	61±0.02	-30±3.1
F11	1	1	361±1.04	56±0.05	-27±1.2

The model validation of each response was carried out at a significance level *P*  <  0.05 and is furnished in Table 3. From the model equations the value of R^2^ reflects the dependency of the factors with the responses.

**Table 3:** Model validation statistics

**Response**	**Suggested fit summary**	***P*** ** value**	**R** ^ 2 ^	**Model equation**
Particle size	2FI	0.0073	0.8029	Particle size = 174.73 + 48.90 SPC + 33.84CH + 60.75 SPC
% Entrapment efficiency	Quadratic	0.0224	0.8827	%EE = 71 - 3.77 SPC - 7.50CH^ 2 ^
Zeta potential	Quadratic	0.0014	0.9631	Zeta-potential = - 37.33 + 1.13CH + 0.000SPC^ 2 ^ + 5.11CH^ 2 ^

**Figure 1:**
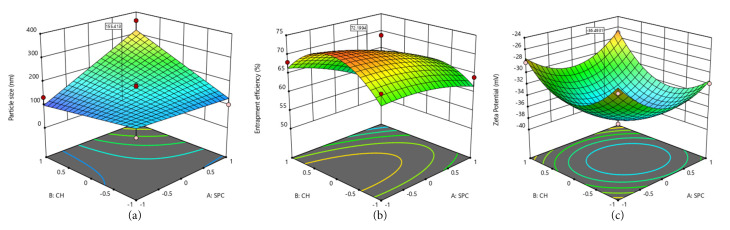
Response surface plot showing the effect of lipids on (a) Particle size, (b) Entrapment efficiency, and (c) zeta potential.

The influence of the factors on the responses are listed in Table 4 and represented by the response surface diagrams in Figure 1. The response surface diagrams in Figure 1 showed that the lipid content greatly affects the particle size and zeta potential, and entrapment efficiency. The model showed that there was no linear correlation between responses and factors. The model proved that the different levels of the factor can produce variation in responses.

**Table 4:** Summary of ANOVA

**Responses**	**Source**	**Sum of squares**	**df**	**Mean square**	**F value**	***P*** ** value**
Particle size	A-SPC	19128.79	1	19128.79	12.67	0.0092 (S)
	B-CH	9163.69	1	9163.69	6.07	0.0432 (S)
	AB	14762.25	1	14762.25	9.78	0.0167 (S)
%Entrapment efficiency	A-SPC	113.57	1	113.57	9.19	0.0290 (S)
	B-CH	14.66	1	14.66	1.19	0.3258 (NS)
	AB	16.00	1	16.00	1.30	0.3067 (NS)
	A^2^	12.71	1	12.71	1.03	0.3571 (NS)
	B^2^	317.65	1	317.65	25.71	0.0039 (S)
Zeta potential	A-SPC	4.83	1	4.83	3.04	0.1419 (NS)
	B-CH	10.22	1	10.22	6.42	0.0523 (S)
	AB	5.76	1	5.76	3.62	0.1155 (NS)
	A^2^	91.96	1	91.96	57.78	0.0006 (S)
	B^2^	147.48	1	147.48	92.66	0.0002 (S)

S, significant; NS, non-significant.

From the above data and the response surface diagram it was found that the lipid composition had a significant effect on the particle size in confirmation with other reported studies.^41^ The entrapment efficiency and zeta potential of the formulation were greatly influenced by the SPC content and presence of CH, respectively.

The numerical optimization yielded an optimized molar ratio of SPC:CH= 1.44:1 with a prediction of particle size– 155.4 nm, %Entrapment efficiency -72.19%, and Zeta potential–(-36.49 mV).

**Figure 2:**
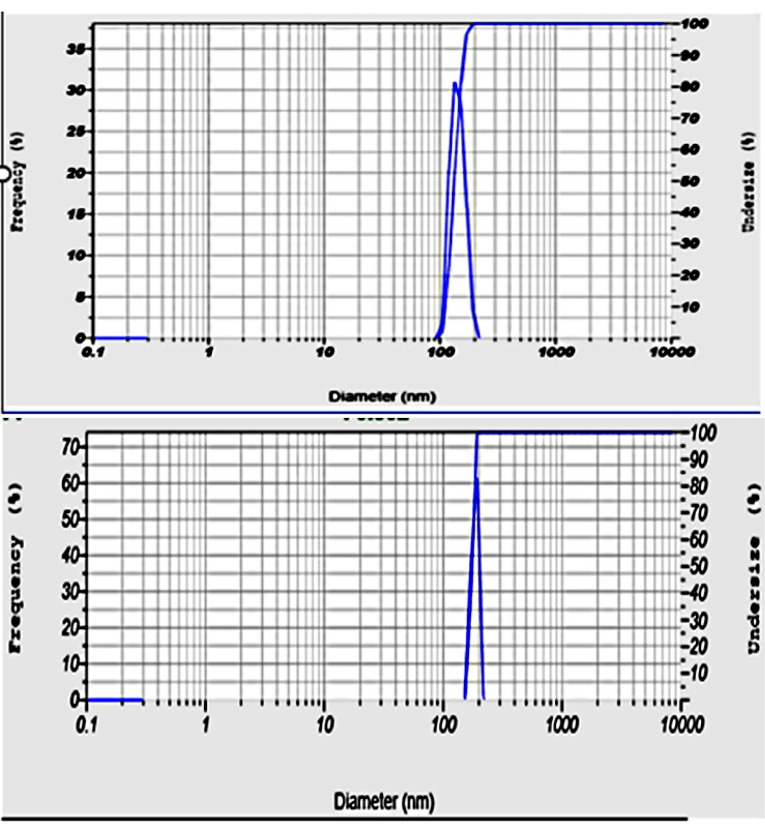
Particle size of the optimized formulation before(a) and after(b) lyophilization.

A trial batch of optimized formulation was prepared using the above molar ratio of SPC and CH by ethanol injection method. The product was evaluated for all the responses. The % prediction error was estimated from the predicted and observed data as listed in Table 5. All the responses showed a % prediction error of less than 10%. Figure 2 represents the particle size of the optimized formulations before and after lyophilization.

**Table 5:** Comparison of predicted and experimental values of the optimized formulation

**Code for responses**	**Responses**	**Predicted**	**Observed**	**%Prediction error**
R1	Particle size (nm)	155.4	144.2±1.21	7.76
R2	% Entrapment efficiency	72.19	70.4±0.05	2.54
R3	Zeta potential (mV)	-36.49	-35±0.67	4.26

* All the observed values are mean ± SD (n=3).

Hence, the optimized formulation met the percentage prediction error in all aspects of the evaluated responses, hence the CCD was found to be effective in predicting the effect of lipid composition on particle size, zeta potential and entrapment efficiency.

### Surface morphology study

**Figure 3:**
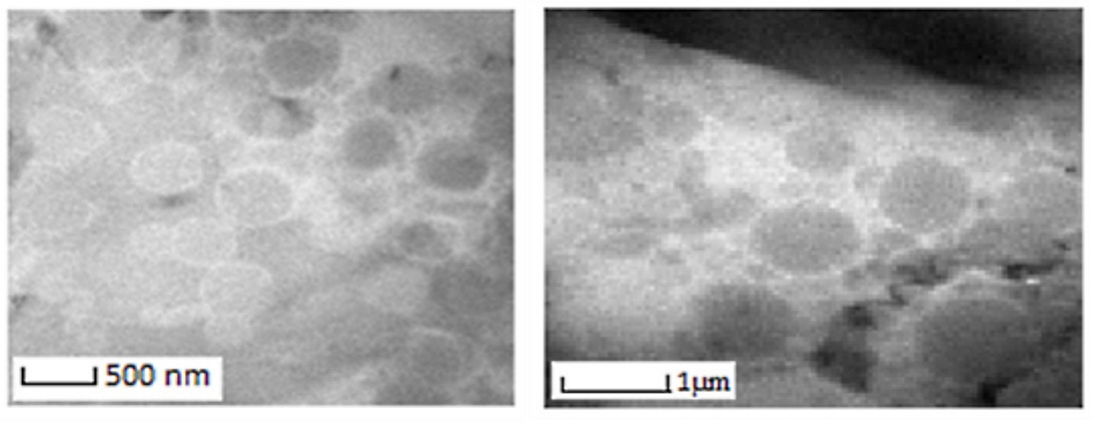
TEM photograph of optimized formulation.

The surface morphology study of the optimized formulation using TEM showed the formation of nanosized vesicles well correlated with the prediction from the optimization process and particle size measurements by dynamic light scattering system, as shown in Figure 3. TEM photographs revealed the liposomes were spherical, and nanosized.^42^

### Atomic force microscopy

**Figure 4:**
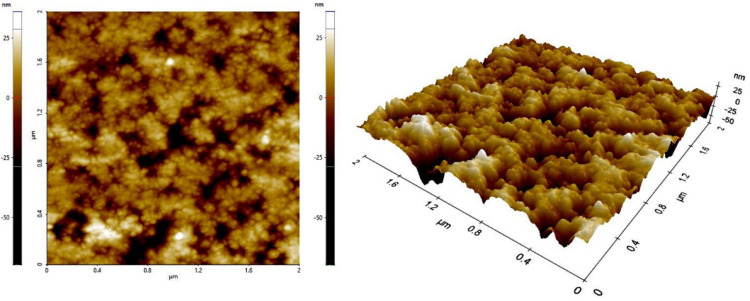
AFM images of optimized formulation.

The AFM images of the aztreonam loaded liposomes further proved the formation of nano sized vesicles as shown in Figure 4.

The particle size of the freeze-dried product was found to be 174±2.32 nm, little more than the predicted responses as shown in Figure 2. The Poly dispersity index (PDI) of the sample was found to be 0.302. The ratio of particle size of the liposomes pre and post lyophilization of all the three trials was found to be 0.83. A value close to one indicates the conservation of nanoparticle size.^43^ The polydispersity index of the optimized formulations after freeze drying was found to be less than 0.5, which indicated a formation of monodisperse system. The surface charge was found to be -34±0.42 mV indicating stability of the drug loaded liposomal powder. Formulation Fopt showed a slight decrease in the entrapment efficiency (69±0.02%) after freeze drying of the product. This slight reduction in entrapment may be due to the leakage of drug from the liposomes during freeze drying.^44^ Therefore, it can be concluded that the freeze-drying condition was successful enough to conserve the nano size of the liposomal aztreonam, and integrity of the nanostructure.

### Differential scanning calorimetry study

**Figure 5:**
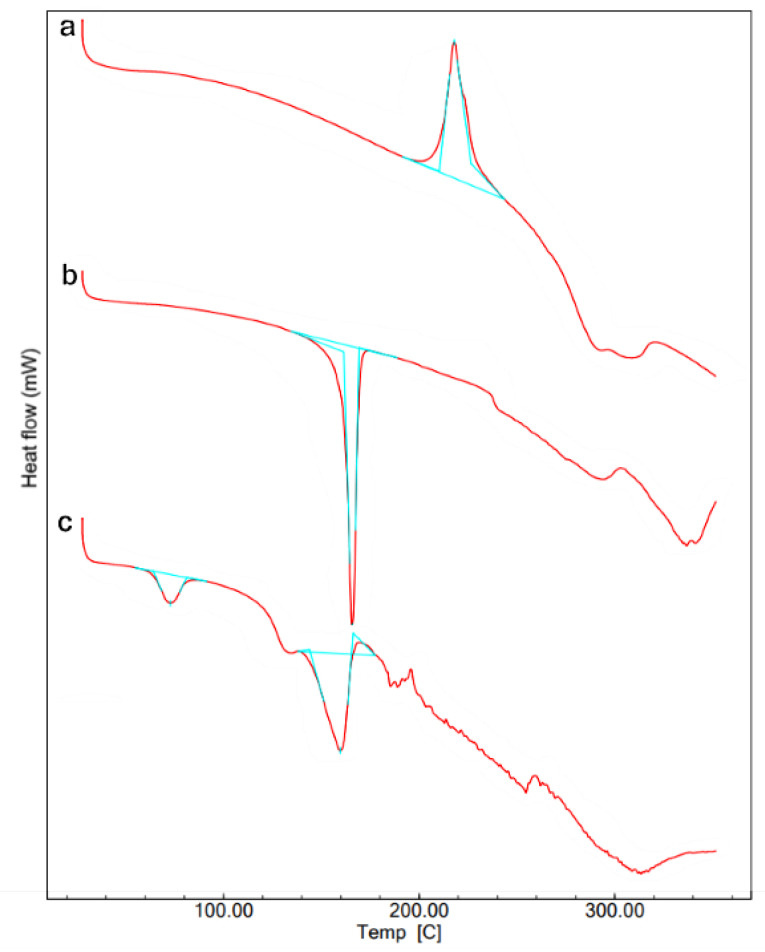
DSC thermograms of formulation F4. (a) Pure aztreonam, (b) Blank optimized liposomal formulation, and (c) Aztreonam loaded optimized liposomal formulation.

The DSC thermograms are shown in Figure 5 indicated the thermal behavior of the drug and the drug loaded liposomal formulations. The pure drug showed an exothermic peak at 218ºC corresponding to its melting with decomposition which correlates with the previous reports of ꞵ polymorph of aztreonam.^45,46^ The empty liposomes showed an endothermic peak at 166.28ºC. The aztreonam loaded liposomes showed the endothermic peak with low intensity at 159ºC. No peak was detected for the pure drug in the formulation it may be due to the reduced crystallinity or conversion of an amorphous form.^26^ The optimized formulation showed the shifting of endothermic peaks. A reduction in the calorimetric measurements (phase transition temperature and heat) indicates the emplacement of the drug inside the lipid bilayer.^42^

### Powder X-ray diffraction study

**Figure 6:**
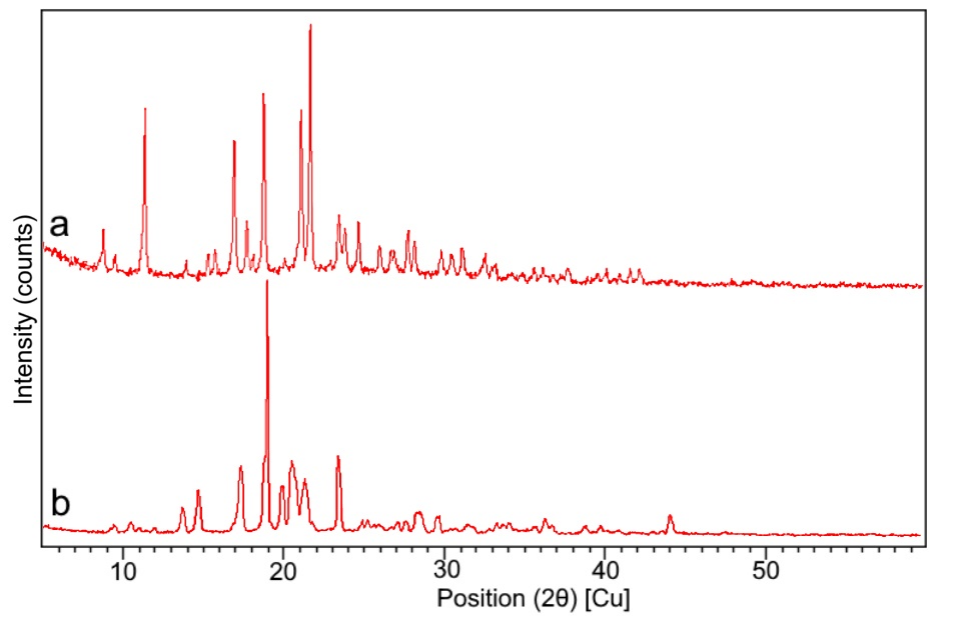
PXRD study. (a) Pure Drug aztreonam, (b) Optimized formulation of liposomal aztreonam.

The PXRD pattern of pure drug exhibited high intensity peaks in the Figure 6a which was in conformity with the thermogram of DSC (Figure 5a) of pure drug revealing its crystallinity. The sharp characteristic peaks of pure drug at 2θ=9º to 30º, reveals ꞵ polymorph of aztreonam, which conformed with the DSC thermogram of the pure drug.^45^ The PXRD pattern of the optimized formulation showed the perseverance of the major peaks of the pure drug with a much lower intensity at the same diffraction angle, which indicated the entrapment of the drug in the lipid matrix.

### Thermogravimetric analysis

**Figure 7:**
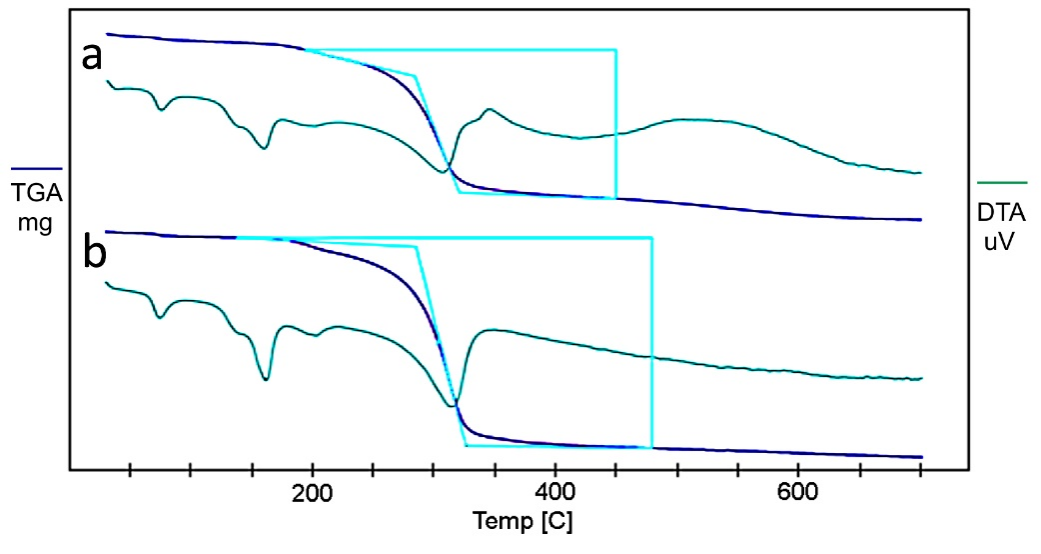
TGA thermograms of freeze-dried optimized formulation. (a) Freshly prepared sample, (b) Sample after 3 months.

TGA was used to measure the physical and chemical changes of the product on thermal analysis. The optimized sample was stored in a glass vial with rubber closure at room temperature at 25°C ± 2°C/65% RH ± 5% RH for three months as per ICH guidelines. The water content of the freeze-dried product was compared with the moisture content of the freshly prepared freeze-dried product by TGA analysis. The residual moisture content and ratio of water loss at a given temperature were determined and expressed in Table 6. The thermograms are shown in Figure 7. The moisture content of the freeze-dried product was found to be less than 5% for the optimized formulation after 3 months of stability study. The TGA revealed that the ratio of water loss was 1.27 and found to be within the limit as per ICH guidelines.^38^ Therefore, the thermal study of the optimized formulation proved the stability of the formulation on storage.

**Table 6:** Determination of residual moisture content

**Optimized sample**	**Weight taken for analysis (mg)**	**Weight observed (mg)**	**% Moisture content**	**Ratio of water loss**
Freshly prepared	2.8	2.712	3.14	-
sample after 3 months	3.8	3.646	4	1.27

### *In vitro* diffusion studies

**Figure 8:**
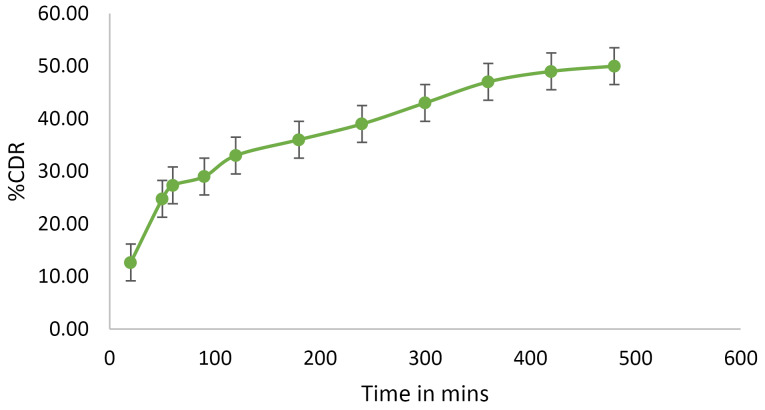
*In vitro* diffusion study of the optimized formulation.

The liposomal solution of the optimized formulation showed a biphasic release of drug in gamble solution as shown in Figure 8. A rapid release of the drug in simulated lung fluid was observed in the first few hours, followed by a sustained release from the optimized liposomal formulation. The* in vitro* release pattern was fitted into various kinetics model and found that the regression coefficient (R^2^) was high for Higuchi release (R^2^= 0.9614) and the Fickian diffusion from the matrix was also confirmed from the release exponent (n value) from Korsmeyer-Peppas model which was found to be n=0.384. It can be concluded from the release studies that the initial burst release, followed by slow release can promote good residence time of the drug in the pleural fluid and thereby better efficacy of the medication.

### Antimicrobial assay

**Figure 9:**
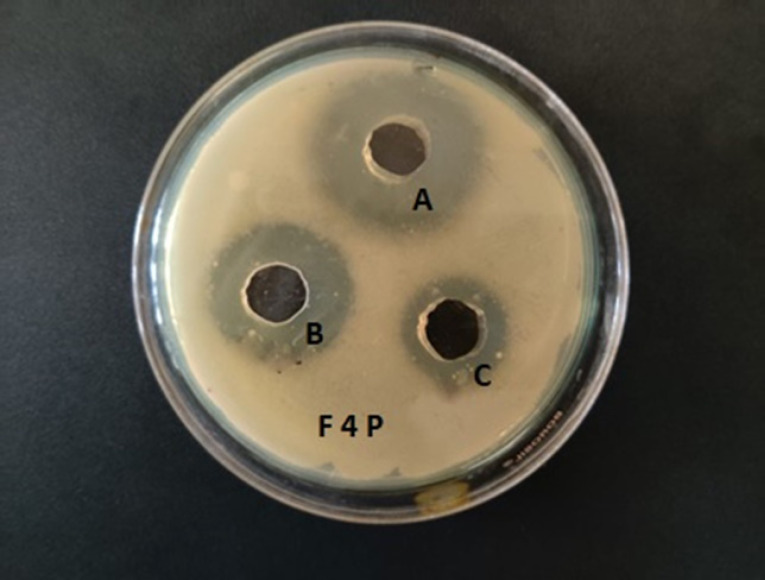
Zone of inhibition study of Formulations Fopt. A represents 100 μg/mL, B represents 50 μg/mL of aztreonam loaded liposomal sample, C represents 50 μg/mL of pure aztreonam.

The minimum inhibitory concentration of the formulation for *P. aeruginosa* was found to be 400 μg/mL. Zone of inhibition for different dilutions of the optimized preparation for the strain is listed in Table 7. Zone of inhibition of the formulation against the bacterium is shown in Figure 9.

**Table 7:** Microbial testing of the drug

**Concentration** **(μg/mL)**	**Zone of inhibition of Fopt (mm)**	**Zone of inhibition of standard aztreonam (mm)**
50	10.32±0.01	8.0±0.05
100	12.39±0.02	

*The results represent mean ± standard error of mean (n=3).

Many studies have proved that liposomal encapsulation of antibiotics increases the efficacy of the drug with reduced side effects.^22,33^ Drug encapsulated in liposomes is safeguarded from the bacterial lactamase or carbapenemase. Earlier scientific literature reported that aztreonam was inactive against gram-positive bacteria but in low concentration much effective against Gram negative bacteria.^47^ A similar observation also resulted in the present study. The MIC of aztreonam against *P. aeruginosa* was ≤8 μg/mL while the MIC of the optimized liposomal formulation was found to be 400 μg/mL.^48^ Therefore, the liposomal aztreonam exhibited a higher antimicrobial effect compared to free drug. The zone of inhibition study also showed a satisfactory result at two different concentrations of the optimized formulation. The liposomal integration with the bacterial cell membrane resulted in high release of the drug into the cells, and hence showed potential antibacterial activity. The prolonged interaction and fusion of the liposomes with the bacterial cell membrane could deliver its content into the cell and thereby could inhibit the cell wall synthesis of the bacteria.^33^ This might constitute the reason for obtaining good zone of inhibition against both the species compared to the pure drug.

### In-vitro cell viability and uptake study

**Figure 10:**
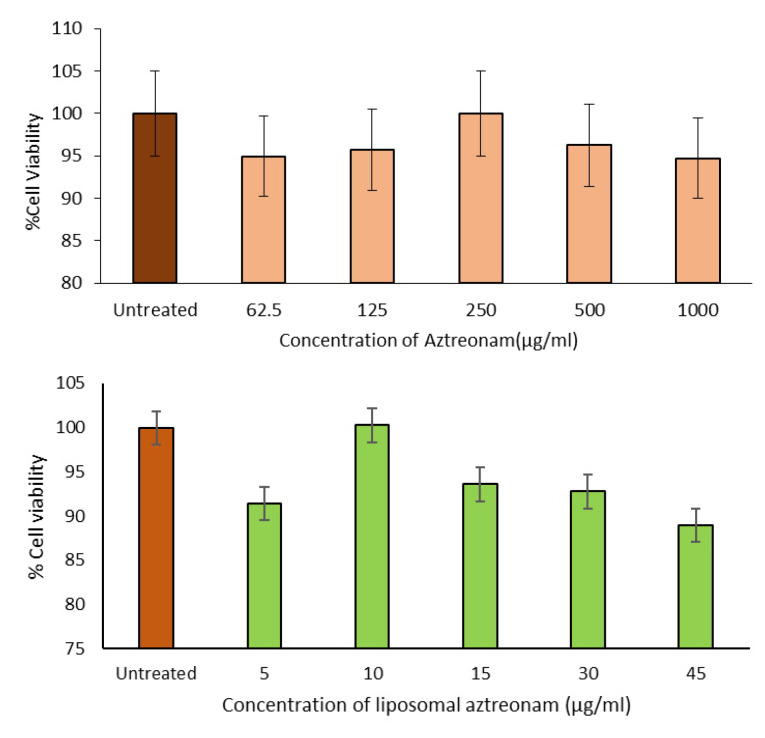
Cell viability study: (a) Pure drug aztreonam, (b) Liposomal aztreonam.

The MTT assay was used to evaluate and estimate the toxic effects and cell viability, respectively on the cell line, after treating with different dilutions of aztreonam and the optimized formulation. The untreated medium was served as a positive control with cell viability of 100%. The percentage viability of the cells on different concentrations of drug and formulation is presented in Figure 10. The results indicated that the epithelial cells could tolerate all the concentrations of liposomal aztreonam. Therefore, it can be concluded that the liposomal formulations were non toxic to the cells. A concentration of 30 μg/mL was selected for cell uptake studies to avoid any toxicity from overly high concentrations of the formulations.^36,49^

**Figure 11:**
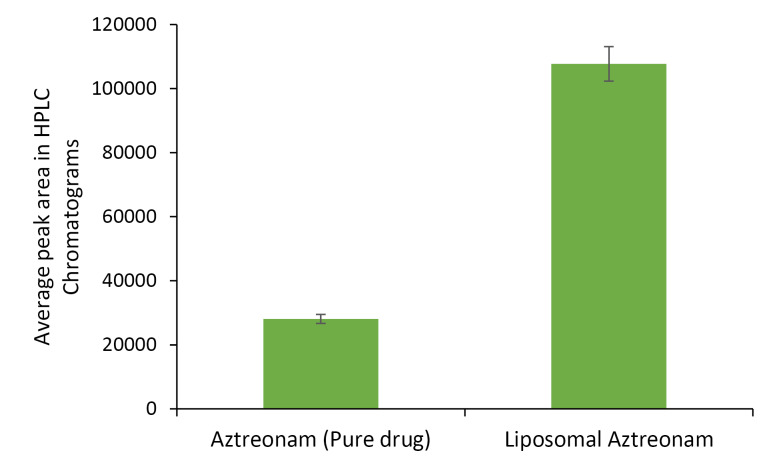
Cell uptake study of aztreonam and liposomal aztreonam.

The study of intracellular uptake of aztreonam from the lipid carrier was performed by incubation of the cells with the free drug and optimized formulation at a concentration of 30 μg/mL for 24 hours. The cells were extracted and analyzed by the developed HPLC technique. The HPLC assay revealed that the percentage uptake of the pure drug was 11.15%± 0.023 while the same for liposomal formulation was 44.36%±0.12 in the epithelial cell. An improved cell uptake of approximately 4 times was observed with the liposomal formulation compared to the pure drug as illustrated in Figure 11. The pure drug has a low log *P* value, therefore the cell uptake study indicated the permeation of the drug was improved significantly in the lipid vesicle. This can be further supported by the surface charge of the formulation and this observation is following the reported studies by Patil et al.^50^ Hence, the penetrability of aztreonam has been improved in a lipid carrier system. The improved penetrability can be utilized in the invasion of biofilm produced during pneumonia.

### Stability study

The stability is a major concern of liposomal formulation to avoid leakage and agglomeration of the particles. The stability study indicated that with time the particle size and zeta potential were increased slightly from 174±2.32 nm to 183±1.32 mm and -34±0.42 mV to -33±0.51 mV respectively while the entrapment efficieny was decreased by 2% as shown in Table 8. Therefore, there were no significant changes in the formulation property at the storage conditions. The stability study for 3 months revealed that the formulation was able to maintain its integrity at room temperature as it did not show any major changes in its physicochemical property.

**Table 8:** Stability study of optimized liposomal aztreonam at 25°C ± 2°C/65% RH ± 5% RH for 3 months

**Time**	**Particle size (nm)**	**Zeta potential (-mV)**	**% Entrapment efficiency**
Freshly prepared	174±2.32	-34±0.42	69±0.02
Sample after 1 month	177±3.01	-34±0.89	68.4±0.09
Sample after 2 months	179±2.64	-33±0.19	68±0.12
Sample after 3 months	183±1.32	-33±0.51	67.12±0.06

*The results represent mean ± standard error of mean (n=3).

Based on these observations it can be concluded that the liposomal aztreonam can be suitable for a novel drug delivery to lungs.

## Conclusion

The present study aimed to evaluate the possibility of liposomal aztreonam formulation for delivery of drugs to the lungs as a target. It was observed that the lipid components had significant effects on the physicochemical properties of liposomes like particle size, zeta potential, and entrapment efficiency. Hence statistical optimization was carried out to predict the attributes of lipids towards the properties of liposomes. The particle size, zeta potential and drug entrapment efficiency of the optimized formulation at a SPC:CH =1.44:1 was found to be optimum for a nano stable delivery system. The polydispersity index was found to be less than 0.5 and indicated a monodisperse system. The sustained *in vitro* release of the drug from the liposomal matrix in simulated lung fluidproved the possibility of increased residence time of the drug in the lungs. The DSC, and PXRD study revealed the encapsulation of the drug in the lipid vitrified matrix. The residual moisture determination study by TGA within a stipulated period proved the stability of the preparation on storage. The accelerated stability studies also revealed the conservation of physicochemical properties of the liposomes at specific storage conditions. The optimized formulation showed the better antimicrobial property and exhibited enhanced cellular uptake of aztreonam in a lipid carrier compared to the pure drug. Therefore, it can be concluded that the liposomal aztreonam is worthy of further study as effective delivery of drug in the treatment of pneumonia by inhalation.

## Ethical Issues

Not applicable.

## Conflict of Interest

The authors declare no conflict of interest.

## Acknowledgments

The authors are grateful to the management and principal of Krupanidhi College of Pharmacy, Bangalore to support the research work by providing excellent laboratory facilities. Authors extend their gratitude to Govt College of Pharmacy and Indian Institute of Science, Bangalore for their help in carrying out antimicrobial and TEM and AFM Studies, respectively.
